# From Phenology and Habitat Preferences to Climate Change: Importance of Citizen Science in Studying Insect Ecology in the Continental Scale with American Red Flat Bark Beetle, *Cucujus clavipes*, as a Model Species

**DOI:** 10.3390/insects12040369

**Published:** 2021-04-20

**Authors:** Radomir Jaskuła, Marta Kolanowska, Marek Michalski, Axel Schwerk

**Affiliations:** 1 Department of Invertebrate Zoology and Hydrobiology, Faculty of Biology and Environmental Protection, University of Lodz, Banacha 12/16, 90-237 Łódź, Poland; 2 Department of Geobotany and Plant Ecology, Faculty of Biology and Environmental Protection, University of Lodz, Banacha 12/16, 90-237 Łódź, Poland; marta.kolanowska@biol.uni.lodz.pl; 3 Department of Biodiversity Research, Global Change Research Institute AS CR, 603 00 Brno, Czech Republic; 4 Department of Experimental Zoology and Evolutionary Biology, Faculty of Biology and Environmental Protection, University of Lodz, Banacha 12/16, 90-237 Łódź, Poland; marek.michalski@biol.uni.lodz.pl; 5 Department of Landscape Art, Institute of Environmental Engineering, Warsaw University of Life Sciences—SGGW, Nowoursynowska 166, 02-787 Warsaw, Poland; axel_schwerk@sggw.edu.pl

**Keywords:** Coleoptera, Cucujidae, North America, USA, Canada, phenological activity, macrohabitat preferences, habitat loss, citizen scientific data, iNaturalist

## Abstract

**Simple Summary:**

Studies of widely distributed species often are problematic as such research usually needs to engage a lot of time, a large team of field workers, and big financial support before good quality data will be collected. Citizen scientists allow to study different aspects of species biology and ecology with significantly reduced basic operational costs of such studies. Based on the data deposited in the iNaturalist database, we studied the ecology of the American flat bark beetle in the entire area of its species range. The results clearly show high value of citizen science, particularly in studies focused on habitat preferences and phenology in both recognized subspecies of this taxon.

**Abstract:**

The American red flat bark beetle, *Cucujus clavipes*, is a wide distributed saproxylic species divided into two subspecies: ssp. *clavipes* restricted to eastern regions of North America and ssp. *puniceus* occurring only in western regions of this continent. Unique morphological features, including body shape and body coloration, make this species easy to recognize even for amateurs. Surprisingly, except some studies focused on physiological adaptations of the species, the ecology of *C. clavipes* was almost unstudied. Based on over 500 records collected by citizen scientists and deposited in the iNaturalist data base, we studied phenological activity of adult beetles, habitat preferences and impact of future climate change for both subspecies separately. The results clearly show that spp. *clavipes* and ssp. *puniceus* can be characterized by differences in phenology and macrohabitat preferences, and their ranges do not overlap at any point. Spp. *clavipes* is found as more opportunistic taxon occurring in different forests as well as in urban and agricultural areas with tree vegetation always in elevations below 500 m, while elevational distribution of ssp. *puniceus* covers areas up to 2300 m, and the beetle was observed mainly in forested areas. Moreover, we expect that climate warming will have negative influence on both subspecies with the possible loss of proper niches at level even up to 47–70% of their actual ranges during next few decades. As the species is actually recognized as unthreatened and always co-occurs with many other species, we suggest, because of its expected future habitat loss, to pay more attention to conservationists for possible negative changes in saproxylic insects and/or forest fauna in North America. In addition, as our results clearly show that both subspecies of *C. clavipes* differ ecologically, which strongly supports earlier significant morphological and physiological differences noted between them, we suggest that their taxonomical status should be verified by molecular data, because very probably they represent separate species.

## 1. Introduction

The citizen science (known also as “online citizen science”, “community science”, and/or “volunteer monitoring”) is often defined as a scientific research conducted, at least in part, by nonprofessional and/or amateur scientists [1,2]. Even if it has a long history in the ecological sciences and has made important contributions to science, education, and society [3,4,5,6], the term appeared relatively recently in the scientific world but very quickly started to play a very important role in the scientific community all over the world. Although different environmental sciences use the data collected by citizen scientists, most probably the highest influence of this activity is observed in studies focused on biodiversity and distribution of fungi, plants, and land animals including insects. In the literature, there are numerous examples where observations by citizen scientists allow to assess, monitor, and predict biodiversity on local, regional, country, continental, or global scale, e.g., [3,7,8,9,10,11,12,13,14]. In many cases citizen scientists helped, e.g., to delimit the geographical distribution of a species, e.g., [15,16]; to find species for the first time in the country, e.g., [17,18,19,20], in the continent, e.g., [21,22,23]; or even to discover new taxa, e.g., [21,24,25,26,27,28,29]. Moreover, such observations allow to monitor migratory birds, e.g., [30,31,32,33,34]; rare and endangered species, e.g., [35,36,37,38]; expansion of pests, e.g., [20,39,40]; or alien and/or invasive species, e.g., [18,22,39,41,42,43,44,45,46,47,48,49,50,51] as well as colonization of new human-made habitats, e.g., [51]. In addition, numerous studies show that data collected by amateur scientists allow to describe new interactions between species, e.g., [17,52], to investigate animal phenology, e.g., [53], and behavior, e.g., [54], to find changes in species abundance and demography, e.g., [55,56,57], and other threats for local fauna and flora being important for nature conservation, e.g., [8,58,59,60,61,62,63,64,65].

Besides many local, regional, or national programs and initiatives focused on biodiversity studies for which help and support by citizen scientists is crucial, numerous online databases or even social media play a very important role in collecting citizen scientific data [66]. Some of these webpages are strictly focused on a single taxonomic group, e.g., eBird.org on birds, AntWeb.org on ants, or BugGuide.Net on insects and other terrestrial arthropods, while others like Zooniverse or iNaturalist accept records of all animals or organisms living on the Earth respectively. Among all mentioned projects, the iNaturalist, with its 37 language versions, over 3.5 million total registered users, and over 66 million observations, is currently the most popular citizen science website. As a consequence, numerous scientific papers based at least partly on these records are published every year, e.g., [8,11,13,14,23,24,27,29,38,39,50,53,59,65,67,68,69,70,71,72].

Although citizen science data play a crucial role in numerous biodiversity and ecological studies, it is important to note that sometimes they are justifiably criticized for selective reporting, uneven sampling, incomplete detection, or—crucial for future use of such data—for species misidentification. The last of the mentioned problems often appears in case of records based on photographs and is observed, e.g., in cases of poor picture quality or resolution or improper orientation of the photographed object, which is especially common in the case of invertebrates including many insects. As a result, usually the best data are available for species which can be easily identified, including taxa characterized by medium and/or big body size, diurnal activity, vivid body coloration, and/or unique body shape as it is often observed in butterflies, dragonflies, or some beetles.

The flat bark beetles (Coleoptera: Cucujidae) are a small insect family with only about 70 species distributed worldwide except Africa, polar regions, and numerous oceanic islands [23,73,74,75,76,77,78,79,80,81,82,83,84,85,86]. Among them, only members of genera *Cucujus* Fabricius, 1775, and *Pediacus* Shuckard, 1839, are known from North America, with the single species, *Cucujus clavipes* Fabricius, 1781, representing the first genus [76,77,86]. The species is recorded from Canada and the USA, and its identification is rather easy because of very characteristic vivid red body coloration, unique strongly flattened body shape, and medium body size. Although currently it is divided into two subspecies (earlier recognized as two separate species), even in the field, their misidentification is rather unlikely as they are geographically separated, with *C. c. clavipes* Fabricius, 1781, known from eastern part of North America and *C. c. puniceus* (Mannerheim, 1843) noted only from western regions of this continent [76,77,86]. In addition to the wide area occupied by this species, both subspecies of *C. clavipes* are not rare or even very common in most of the species range, which together with very attractive body coloration, make this beetle a common object of field observation, including citizen scientists using their camera to photograph nature. All these features make *C. clavipes* a perfect model for studies focused on insect ecology when help and support of citizen science is planned to be used.

The aim of this paper is to evaluate the role of citizen science, particularly observations done using the iNaturalist website (iNaturalist.org), in studying selected aspects of insect ecology with the American red flat bark beetle, *Cucujus clavipes*, as a model taxon, including (1) adult phenological activity of its both subspecies distributed in western and eastern parts of North America, (2) habitat preferences of both subspecies, (3) present and future species range for its both subspecies in the context of global climate change. As far as we know, the study is the first case when citizen science is used to study present and future ecology of insect species at the continental scale.

## 2. Materials and Methods

### 2.1. Sampling Citizen Scientific Data from iNaturalist

**Figure 1:**
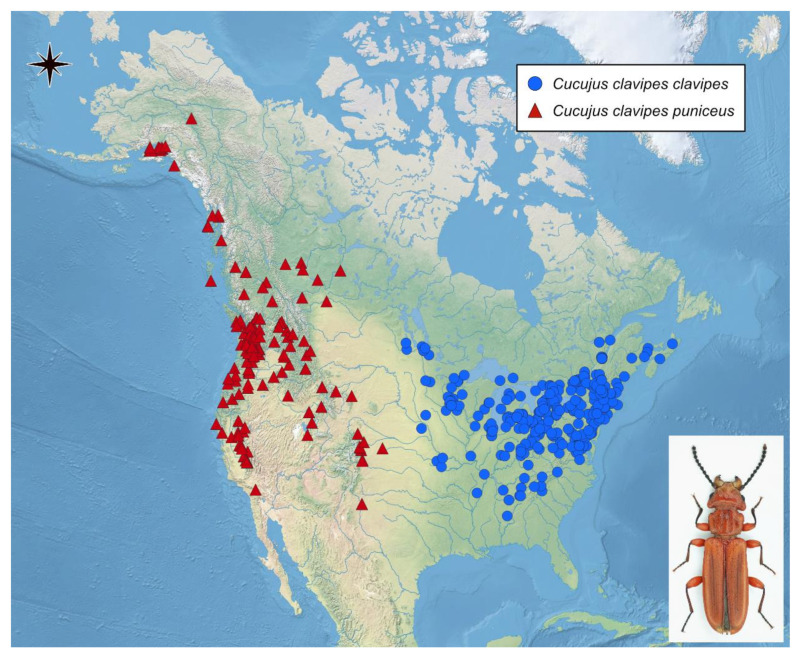
Location of Cucujus clavipes records based on observation by citizen scientists published on iNaturalist.org and used in this study (picture shows spp. clavipes; phot. M. Michalski.

Although numerous records for *Cucujus clavipes* are known from the literature [76,77,86,87,88,89,90,91,92,93,94,95,96,97,98,99,100,101,102,103,104] and various online resources, such as Global Biodiversity Information Facility (www.gbig.org, accessed on 31 August 2020), all data used in this study were taken directly and only from the iNaturalist data base (www.inaturalist.org, accessed on 31 August 2020). All pictures of American Cucujidae published in the database since its beginning to the end of August 2020 were checked for records of *C. clavipes*. Although the species can be easily recognized based on photographs, to avoid any potential misidentification, we excluded all records with poor picture quality or resolution and/or insufficient orientation of the photographed beetles. We also excluded observations lacking detailed GPS collecting data. As a result, almost 600 pictures were verified of which a few dozen were excluded. Finally, in total, 548 records including 368 for ssp. *clavipes* (observations from 1986 to 2020) and 180 for spp. *puniceus* (observations from 2009 to 2020) were amassed (Figure 1, Data S1). For all these data locality, date of observation and GPS coordinates were collected.

### 2.2. Phenology of Adults

As almost all records published in the iNaturalist database include photographs of only a single individual, each observation by citizen scientist was accepted as a single record. To examine any differences in phenological activity between the subspecies of C. *clavipes*, which potentially would be supported by their geographical separation, ssp. *clavipes* and ssp. *puniceus* were analyzed separately. In this analysis we accepted only records of adult beetles and with full date of observation.

### 2.3. Habitat Preferences

To analyze elevational distribution of the *Cucujus clavipes* subspecies the original CSV file downloaded directly from the iNaturalist, including GPS coordinates of each record, was converted into ESRI shapefile (*.SHP) using online MyGeodata software by the GeoCzech, Inc. (https://mygeodata.cloud/converter/csv-to-shp, accessed on 1 September 2020) and accepting World Geodetic System WGS 84 (EPSG:4326) as coordinate system. As a source of altitude data, we used a GeoTIFF raster file derived from SRTM15+ project. Horizontal resolution of the raster was 15 Arc Sec what corresponds to 0.5 × 0.5 km on the equator while accuracy of altitude was 1 m [105]. The GeoTIFF raster file was taken from the Open Topography (portal.opentopography.org, accessed on 17 February 2021). To connect GPS coordinates to altitudinal data, the function “sample raster values” of QGIS v.3.10, was used (QGIS.org, accessed on 17 February 2021). Graphs illustrating *Cucujus clavipes* altitudinal distribution were prepared with R v.4.0.3 [106] with ggplot2 v.3.3.3 library [107].

To analyze macrohabitat preferences of the *Cucujus clavipes* subspecies, the Global Land Cover by National Mapping Organizations ver. 3 (GLCNMO; https://globalmaps.github.io/glcnmo.html, accessed on 10 December 2020) was used.

In order to study if variation in environmental parameters on the plots reflects the presence of the subspecies, a Principal Components Analysis (PCA) was carried out using Canoco for Windows 4.56 [108,109]. The environmental variables: solar radiation in December (SRD), which is the month with the shortest days; solar radiation in June (SRJ), which is the month with the longest day; mean annual temperature (°C) (MAT); maximum temperature of the warmest month (°C) (MTWM); minimum temperature of the coldest month (°C) (MTCM); annual precipitation (mm) (AP); and altitude above sea level (m) (ASL) were included in this analysis. These data were downloaded from WorldClim v. 2.1 with the resolution of 10 arc-minutes based on GPS coordinates of each record. As these variables were measured in different units, centering and standardization were applied and the data were log transformed (log(y + 1), because of zero values).

### 2.4. Impact of Climate Change

**Figure 2:**
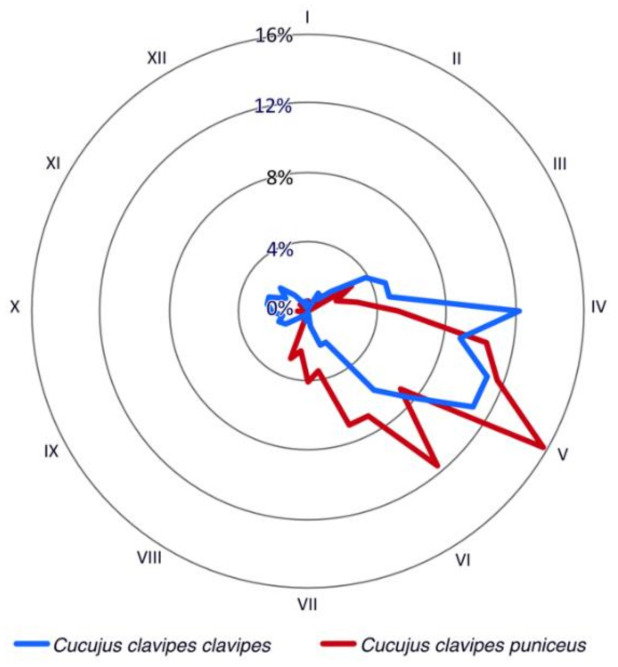
Phenological activity of adult *Cucujus clavipes* subspecies (particular months of the year are written as Roman numerals).

Gathered records of *C. clavipes clavipes* (368 records) and *C. c. puniceus* (180 records) were rarified using 5 classes of habitat heterogeneity and a minimum distance of 10 km as calculated in SDMtoolbox 2.3 for ArcGIS [110]. The final database of localities included 90 records of ssp. *clavipes* and 84 of subsp. *puniceus* (Figure 2, Data S1).

The ecological niche modelling was done using the maximum entropy method in MaxEnt version 3.3.2 [111,112,113] based on presence-only observations of the studied subspecies. For the modelling bioclimatic variables in 10 arc-minutes of interpolated climate surface downloaded from WorldClim v. 2.1 [114] were used. Eleven of 19 variables were removed from the analyses due to their high correlation (above 0.9) as calculated in Pearson correlation coefficient (Table 1) computed using SDMtoolbox 2.3 for ArcGIS [110]; only the following eight were used: bio1 (annual mean temperature), bio4 (temperature seasonality (standard deviation ×100)), bio6 (min temperature of coldest month), bio9 (mean temperature of driest quarter), bio10 (mean temperature of warmest quarter), bio11 (mean temperature of coldest quarter), bio12 (annual precipitation), bio15 (precipitation seasonality (coefficient of variation)). The other variables used in modelling of current potential range of the studied subspecies were: (1) solar radiation (srad 1–12 (solar radiation in particular month, starting with 1 for January and ending with 12 for December)), (2) soil class (0—acrisols, 1—albeluvisols, 2—alisols, 3—andosols, 4—arenosols, 5—calcisols, 6—cambisols, 7—chernozems, 8—cryosols, 9—durisols, 10—ferralsols, 11—fluvisols, 12—gleysols, 13—gypsisols, 14—histosols, 15—kastanozems, 16—leptosols, 17—lixisols, 18—luvisols, 19—nitisols, 20—phaeozems, 21—planosols, 22—plinthosols, 23—podzols, 24—regosols, 25—solonchaks, 26—solonetz, 27—stagnosols, 28—umbrisols, and 29—vertisols), and (3) land cover (1—broadleaf evergreen forest), 2—broadleaf deciduous forest, 3—needleleaf evergreen forest, 4—needleleaf deciduous forest, 5—mixed forest, 6—tree open, 7—shrub, 8—herbaceous, 9—herbaceous with sparse tree/shrub, 10—sparse vegetation, 11—cropland, 12—paddy field, 13—cropland/other vegetation mosaic, 14—mangrove, 15—wetland, 16—bare area, consolidated (gravel, rock), 17—bare area, unconsolidated (sand), 18—urban, 19—snow/ice, 20—water bodies). 

The data on distribution of soil classes were obtained from Global Soil Information [115] (http://www.soilgrids.org, accessed on 20 December 2020) with a 250 m^2^ resolution and upscaled to fit the resolution and extent of the bioclimatic variables. The data on solar radiation in each month were downloaded from WorldClim v. 2.1 with the resolution of 10 arc-minutes. The Global Land Cover by National Mapping Organizations ver. 3 (GLCNMO) was a source of information about land cover in ENM analyses. 

Because some previous studies [116] indicated that usage of a restricted area in ENM analysis is more reliable than calculating habitat suitability on the global scale, the area of the analysis was restricted to 72.91–23.41° N–168.58–48.92° W. Predictions of the future extent of the climatic niches of studied insect in 2080-20100 were made using climate projections developed by CNRM/CERFACS modelling group for Coupled Model Intercomparison Project (CNRM-CM6-1) for four Shared Socio-economic Pathways ([117] SSPs): 126, 245, 370 and 585. These pathways are trajectories adopted by the Intergovernmental Panel on Climate Change (IPCC). The scenarios offer a broader view of a “business as usual” world without future climate policy, with global warming in 2100 ranging from a low of 3.1°C to a high of 5.1°C above pre-industrial levels.

In all analyses, the maximum number of iterations was set to 10,000 and convergence threshold to 0.00001. The neutral (=1) regularization multiplier value and auto features were used. All samples were added to the background. The “random seed” option which provided a random test partition and background subset for each run was applied. Twenty percent of the samples were used as test points. The run was performed as a bootstrap with 100 replicates, and the output was set to logistic. In this analysis, all operations on GIS data were carried out using ArcGis 10.6 (Esri, Redlands, CA, USA). Additionally, to avoid dubious projections, the *“fade by clamping”* function in *MaxEnt* was enabled. This precluded extrapolations outside the environmental range of the training data [118]. The evaluation of the created models was made using the area under the curve AUC; [119,120] and True Skill Statistic TSS; [121].

SDMtoolbox 2.3 for ArcGIS [110] was used to visualize changes in the distribution of suitable niches of the studied subspecies caused by the global warming. To compare distribution model created for current climatic conditions with future models all SDMs were converted into binary rasters and projected using the Goode homolosine as projection. The presence threshold was estimated based on the median values of grids in which studied species occur in models created using present-time—0.48 for *C. c. clavipes* and 0.52 for *C. c. puniceus*.

## 3. Results

### 3.1. Activity of Adult Beetles of Cucujus clavipes and C. v. puniceus

Citizen scientific data show that adult beetles of both *Cucujus clavipes* subspecies were observed mainly during the spring period (Figure 1). For *C. c. clavipes,* the highest beetle activity was noted between the end of March/beginning of April and the beginning of May while for *C. c. puniceus* it was observed between mid-April and mid-June. In case of *C. c. clavipes,* the second, significantly smaller peak, was noted also between the end of September until mid-October (Figure 2).

### 3.2. Environmental Parameters vs. Present Distribution of Cucujus clavipes Subspecies

**Figure 3:**
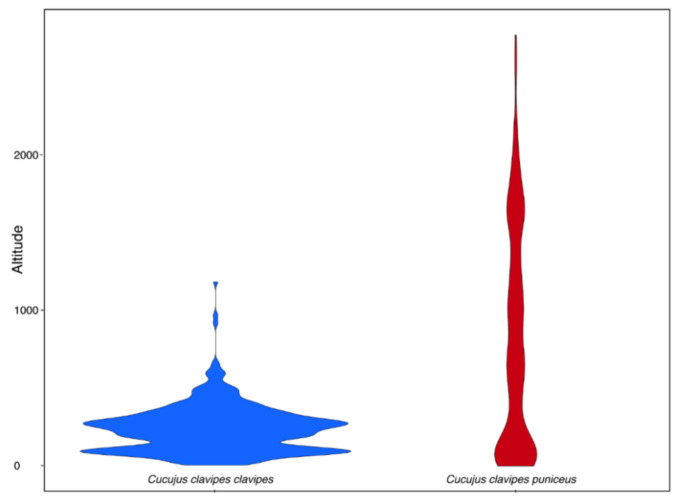
Elevational distribution of *Cucujus clavipes clavipes* (*n* = 368) and *C. c. puniceus* (*n* = 180).

**Figure 4:**
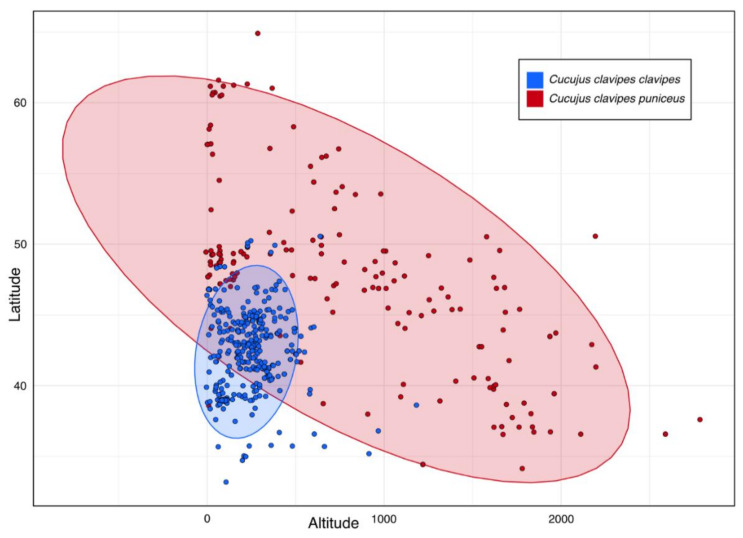
Distribution of *Cucujus clavipes clavipes* and *C. c. puniceus* along elevational and geographic gradients. Elipses include 95% of localities.

The subspecies of *Cucujus clavipes* differ significantly according to elevational distribution. *C. c. clavipes* was found as lowland-highland species with 95% of localities located from the sea level up to 500 m a.s.l. while *C. c. puniceus* can be characterized by wider elevational range with 95% of localities placed from the sea level up to 2300 m a.s.l. (Figure 3 and Figure 4). In addition, *C. c. clavipes* has narrower geographical range and occupies areas between 30° and 50°N while *C. c. puniceus* can be found mainly between 30° and 63°N (Figure 4).

**Figure 5:**
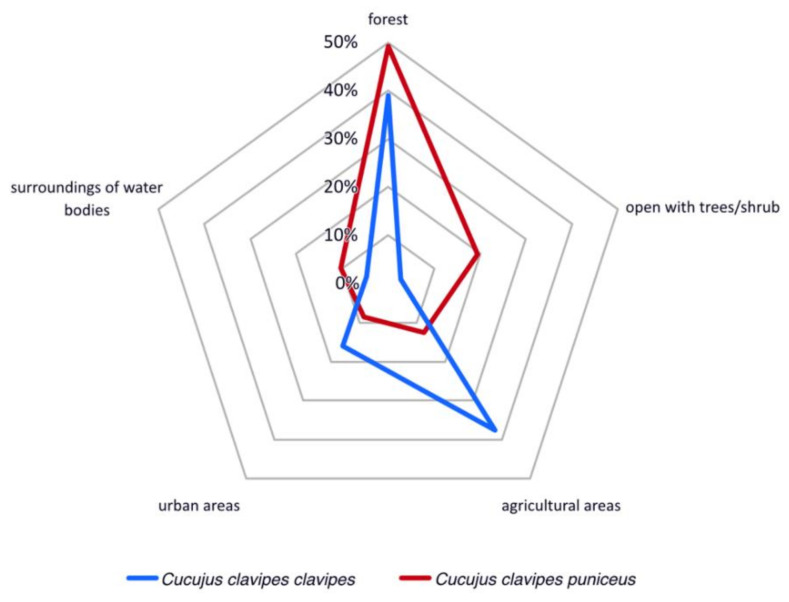
Macrohabitat preferences of *Cucujus clavipes* subspecies based on citizen scientific data.

Analyses of observations made by iNaturalist citizen scientists show that both subspecies of *Cucujus clavipes* can be found in various types of macrohabitats including both natural and disturbed areas. In case of *C. c. clavipes* about 40% of records come from forests while almost 50% from habitats changed by human activity, including agricultural areas where different tree species are growing (ca. 40%) and urban places (ca. 10%). In contrast about 50% records for *C. c. puniceus* come from various forest types, 10% from open areas with tree and shrub species while agricultural and urban areas are not preferred by this subspecies (Figure 5).

**Figure 6:**
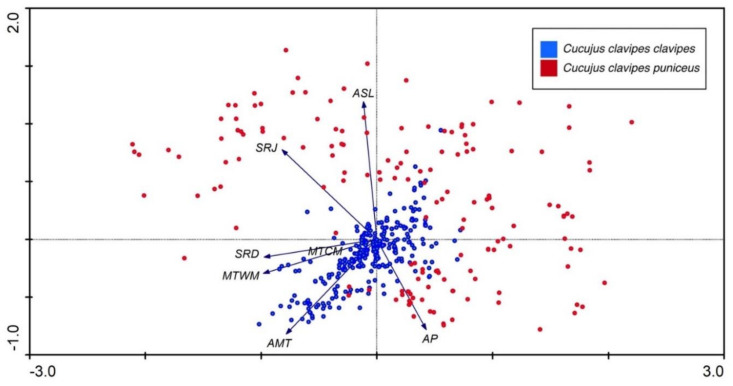
Results of PCA analysis for both subspecies of *Cucujus clavipes*. Environmental symbols: AMT—annual mean temperature, AP—annual precipitation, ASL—altitude above sea level, MTCM—minimum temperature of coldest month, MTWM—maximum temperature of warmest month, SRD—solar radiation in December, SRJ—solar radiation in June.

The Principal Components Analysis made for environmental variables shows that the first and second ordination axes of the PCA (Figure 6) explained 43.0% and 24.2% of the variation in the dataset, respectively. Sites occupied by *C. clavipes clavipes* are more tightly distributed and located in the center and the bottom left part of the PCA diagram, whereas those occupied by *C. c. puniceus* are more loosely scattered and located mainly in the remaining three quarters of the diagram. The former are more positively correlated with annual mean temperature (AMT), mean temperature in the warmest month (MTWM), solar radiation in December (SRD), and mean temperature in the coldest month (MTCM) when compared to the latter. Some sites occupied by ssp. *puniceus* are positively correlated with solar radiation in June (SRJ) and altitude above sea level (ASL) and annual precipitation (AP), respectively.

### 3.3. ENM—Models Evaluation, Limiting Factors and Range Overlap between Subspecies and Impact of Global Warning

Both models of current distribution of suitable niches of the studied subspecies received high scores of both AUC (subsp. *clavipes*—0.983, subsp. *puniceus*—0.967) and TSS (*C. c. clavipes*—0.923, *C. c. puniceus*—0.852) statistics which indicates high reliability of the analyses.

**Figure 7:**
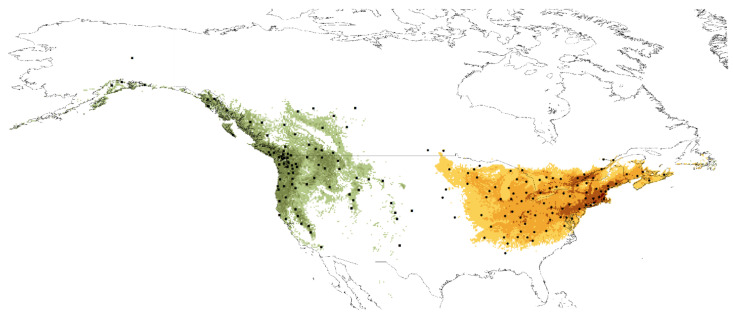
Current distribution of suitable niches of *Cucujus clavipes clavipes* (orange-brown) and *C. c. puniceus* (green) and localities used in ENM analyses (dots—subsp. *clavipes*, squares—subsp. *puniceus*).

As calculated in ENMTools, the ranges of both subspecies do not overlap at any point (Figure 7).

The annual mean temperature (bio1) was the crucial variable influencing models of distribution of the studied insects (Table 2). Both taxa differ, however, in other factors shaping their ranges (Table 2).

### 3.4. Impact of Global Warming

**Figure 8:**
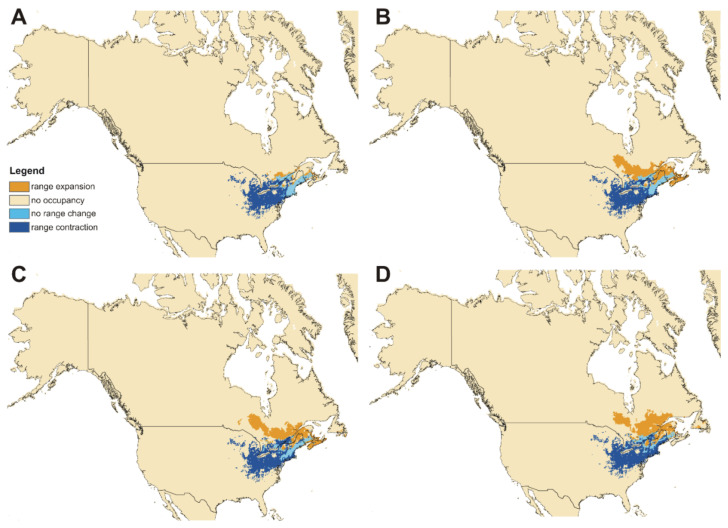
Future changes in the distribution of suitable niches of *Cucujus clavipes clavipes* according to ssp126 (**A**), ssp245 (**B**), ssp370 (**C**), ssp585 (**D**) climate change scenarios.

**Figure 9:**
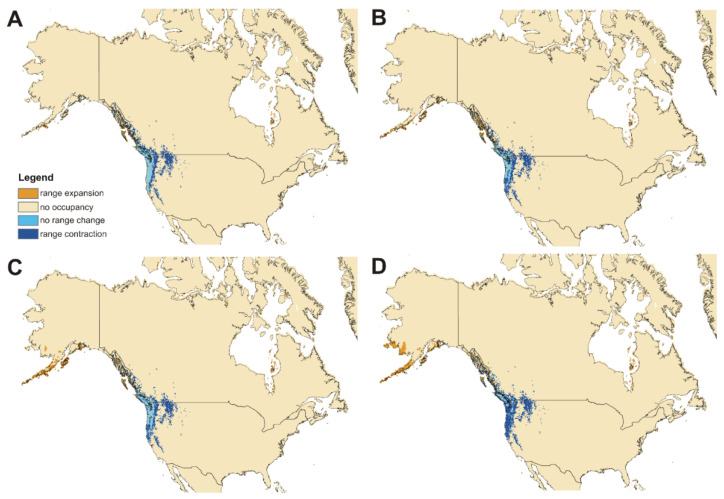
Future changes in the distribution of suitable niches of subsp. *puniceus* according to ssp126 (**A**), ssp245 (**B**), ssp370 (**C**), ssp585 (**D**) climate change scenarios.

As a result of global warming, both studied subspecies will face significant habitat loss (Figure 8 and Figure 9, Table 3). For ssp. *clavipes,* the most damaging will be ssp126 scenario in which the predicted loss of suitable niches will be 70.13%. In the best-case scenario (ssp370) this taxon will lose 26.65% of niches. Most of habitat loss will be observed in the western part of the current geographical range of the subspecies. For ssp. *puniceus,* the most damaging will be ssp585 scenario in which the predicted loss of suitable niches will be 47.10%. The loss will be observed mostly in the southern and eastern part of the subspecies’ range. In the best-case scenario (ssp370) this taxon will lose 26.42% of niches.

Both subspecies will also gain some niches in the areas where currently climatic conditions are not suitable for them. *C. c. clavipes* will have a chance to migrate north-eastern from the current range while new areas for ssp. *puniceus* will be available mostly along the Alexander Archipelago and southern Alaska Peninsula.

## 4. Discussion

### 4.1. Phenological Activity of Cucujus clavipes Subspecies

Although both taxa currently considered as subspecies of *Cucujus clavipes* were described over 170 years ago [86,87] and there are at least several papers providing diverse data for them [76,77,86,87,88,89,90,91,92,93,94,95,96,97,98,99,100,101,102,103,104], phenological activity of these subspecies was never studied. Our paper provides the first comprehensive phenological data for both *C. c. clavipes* and *C. c. puniceus* from the entire area of their ranges. Moreover, this is the only such study for Cucujidae of North America. In addition, until now, only one more species, *C. cinnaberinus* (Scopoli, 1763), was studied according to phenological activity, but in contrast to our paper, the data for this European species were analyzed only for some small parts of species range, particularly for populations from central Europe [122,123].

Although both subspecies of *Cucujus clavipes* are active during the spring period, the phenological activity of ssp. *clavipes* and spp. *puniceus* is different. The eastern one, ssp. *clavipes*, which occurs only up to 500 m a.s.l., starts its activity about two weeks earlier compared to the western ssp. *puniceus* which is known from higher elevations even up to 2300 m a.s.l. Moreover, in the “mountain” spp. *puniceus,* the main peak of its phenological activity ends about half of month later than it is observed in the ”lowland” spp. *clavipes*. Such differences in phenological activity of both subspecies can be clearly explained when we note that insects are poikilothermic animals, and their activity strongly depends on the temperature of the surrounding environment, e.g., [124,125], while the average temperature in the temperate climate zone is followed by length of total day and is strongly correlated with altitude. Although different biotic and abiotic factors should be taken into consideration, there are numerous studies showing that mountainous insect species tend to start their activity later than their lowland relatives, which is especially well observed in vernal taxa, e.g., [126,127,128,129,130,131,132,133,134].

Except for the main peaks of phenological activity of adult beetles, a smaller one was noted in the “lowland” spp. *clavipes*. Although more data about its life cycle and life span are needed, most probably the “second peak” is a result of activity of beetles that finished the life cycle during the summer period and occasionally left their shelters under the bark, most probably because of unexpected higher temperature during the autumn. Similar observations are known for single specimens of European *Cucujus* species [122,135]. On the other hand, we cannot exclude that the autumn peak in the phenological activity of *C. c. clavipes* is connected to the climate warming as similar changes in insect activity/phenology have been noted in many species all around the world, e.g., [136,137,138,139,140,141,142,143,144,145,146,147,148,149].

### 4.2. Habitat Preferences of Cucujus clavipes

Although it is known that *Cucujus clavipes clavipes* occurs in the eastern regions of North America, from Alaska to California, while *C. c. puniceus* occupies western parts of this continent, from Quebec to Alabama [76,77], and both taxa can be characterized by significant differences in physiological adaptations [90,91,95,99,100,101,102,103], and as a consequence need to be characterized by different preferences according to habitat parameters at least in some aspects, surprisingly, no study has been focused on habitat preferences of these beetle taxa. Our study, thanks to the citizen scientific data deposited in the iNaturalist database, allowed to fill this gap and find that ecological niches of *C. clavipes* subspecies do not overlap even if sometimes both taxa can be found in similar types of macrohabitats. *C. c. clavipes* was found to be a subspecies characterized not only by smaller geographic range, but also its altitudinal distribution is significantly narrower in compare to *C. c. puniceus* (Figure 3, Figure 4 and Figure 7). On the other hand, *C. c. puniceus* seems to prefer mainly various forests, and such types of habitats are known for this subspecies from the literature [92,93,96], which can suggest higher habitat specialization in this taxon. At the same time, it can be characterized by being significantly less specific according to studied abiotic factors including, e.g., annual precipitation or annual temperature when compared to *C. c. clavipes* (Figure 6). However, this result can be explained by the wider geographical and particularly wider altitudinal distribution of *C. c. puniceus*, because climatic parameters of a given site should depend to a high degree on its altitude. Accordingly, sites of *C. c. clavipes*, which are largely restricted to elevations up to 500 m a.s.l., are positively correlated with mean annual temperature and mean temperature in the warmest month. Surprisingly, *C. c. clavipes* seems to be a much more opportunistic taxon found not only in different types of forests but also in open areas or even in anthropogenically transformed macrohabitats as agricultural areas or urban areas with tree and/or tree/shrub vegetation. It is interesting that literature data from Nova Scotia, Canada, suggest high habitat specialization of this subspecies as it was found only in coniferous stands in old-growth (120+ years) forests [97].

Although we were able to provide some data about macrohabitat preferences of both *Cucujus clavipes* subspecies, it was not possible to analyze microhabitat data for these taxa including tree species and the dimensions of tree trunks inhabited by larvae. In the literature, one can find that all stages of *Cucujus clavipes* are recorded under the bark of different coniferous and deciduous tree species [88,94,150,151] but with no details about diameter and length of dead tree. At least some of those factors, which are recognized as very important in other *Cucujus* species, e.g., [152,153,154,155,156,157,158,159,160], possibly can be crucial also in case of *C. clavipes*.

### 4.3. The Future of Cucujus clavipes Because of Climate Change

The high anthropogenic impact on biodiversity is the fact and rapid and intense environmental changes due to the human activity are currently observed in almost all of ecosystems all over the world, causing species extinction rates to be the highest in the history of Earth [161,162,163]. The global climate change is recognized as one of the most important factors having negative influence on species diversity, distribution, changes in phenological activity, decline of rare and endangered species or invasion of pests and/or alien taxa, e.g., [164,165,166,167,168,169,170,171,172,173,174,175,176].

In the present paper, we used the Ecological Niche Modelling approach to estimate possible changes in the distribution of the suitable niches of widely distributed and actually unthreatened North American saproxylic beetle *Cucujus clavipes*, checking separately future changes in the ranges of both recognized subspecies. This method has become one of the most important tools for the assessment of the impact of climatic change and has been successfully used, e.g., to evaluate how past climates affected species’ distributions, e.g., [177,178,179,180] or what will be the effects of future climate changes on species, e.g., [181]. Moreover, it helped to map the future distribution of rare species and/or high modelled species richness allowing to appropriately prioritize conservation areas for the establishment of new protected areas, e.g., [182,183,184,185]. In addition, the ENM allowed to determine the potential distribution of species and indicate promising areas for new surveys, e.g., [186,187,188], to define the distribution of recently described taxa and areas where such species may be found, e.g., [189,190], and/or to indicate areas that are suitable for exotic invasive species to establish new populations, e.g., [191,192,193,194,195]. Our results clearly show that ranges of both subspecies of *Cucujus clavipes* will significantly change, and depending on scenario, loss of suitable niches for ssp. *clavipes* will be between 27% and 70% while for ssp. *puniceus* such values are expected at the level of 26–47%. At the same time, it is necessary to mention that spp. *clavipes* will have a chance to migrate in north-eastern direction from the current range while new areas for ssp. *puniceus* will be available mostly along the Alexander Archipelago and southern Alaska Peninsula. Although the process of habitat loss because of future climate change will surely depend on the combination of different environmental factors, the expected north directions of possible future migrations in *Cucujus clavipes* subspecies clearly suggest a crucial role of temperature. Moreover, the importance of global warming can also explain the lower values of habitat loss noted for spp. *puniceus*. This subspecies, compared to ssp. *clavipes*, can be characterized by wider geographical and particularly wider altitudinal distribution (it actually occurs up to 2300 m while spp. *clavipes* is found predominantly below 500 m). As a result, the expected speed of habitat loss caused by the global warming is significantly slower for spp. *puniceus* as potentially it has a chance to migrate in the upper parts of the mountains. In contrast, in ssp. *clavipes* such a strategy can be very limited as the mountain ranges occupied by this taxon are significantly lower. None of the *Cucujus clavipes* subspecies is protected or listed as threatened in the USA and Canada [76], which can suggest stability of their populations and rather low impact of direct human activities on habitats occupied by these taxa including logging and forest management. On the other hand, high values of possible loss of suitable niches expected as a result of climate warming for both subspecies only during next few decades clearly suggest that we should pay more attention not only to endangered and/or protected taxa but also to common and wide-distributed species as it cannot be excluded that in near future they will need special conservation support [196]. *C. clavipes* seems to be a good example of species which probably will start to be threatened in the near future. We need to remember that this taxon is a part of a complex environment with hundreds or even thousands of species co-occurring in the same habitats. Although it is not possible to estimate the total number of such taxa co-occurring with ssp. *clavipes* and spp. *puniceus* along their entire ranges, one can find that only in one relatively small studied area in Alberta, Canada, *C. clavipes puniceus* was found to coexist, depending on the sampling period, together with 234–347 other beetle species and over 2000 other arthropod taxa [92,96]. Very similar results were found in southwestern Nova Scotia, Canada [97], where *C. c. clavipes* was noted together with 345 other beetle species. As particular subspecies can be found in great part of North America, from Alaska to California in the west, and from Quebec to Alabama in the east, and moreover, the ecological niches of both subspecies do not meet, even a cautious estimation suggests that dozens of thousand species occur together with *C. clavipes* in its entire species range.

Currently spp. *clavipes* and spp. *puniceus* are recognized as subspecies of *Cucujus clavipes,* but originally, they were described as separate species [86,87], which was accepted until the end of 19th century [197,198,199]. As it was mentioned above and based on calculation in ENMTools supporting earlier data [76], the ranges of *C. clavipes* subspecies do not overlap at any point. Moreover, literature data show that they significantly differ in morphology of adults and larvae [77] and in physiological adaptations [90,91,95,99,100,101,102,103], while our results prove that they can be characterized also by differences in macrohabitat preferences, phenological activity, and sensitivity for climate change. All these facts strongly suggest that taxonomical status of ssp. *clavipes* and ssp. *puniceus* should be verified by molecular data as most probably they represent separate species.

## 5. Conclusions

Studies focused on ecology of even a single species within its entire species range, especially if it is widely distributed, usually require a lot of time, big financial support for the field work as well as a large team of field workers who can collect necessary data for future analysis. With the help of citizen scientists, most of these requirements disappear or are significantly reduced at least in some types of ecological studies and in case of species which can be easily identified even by amateurs. Our study clearly suggests that the American flat bark beetle, *Cucujus clavipes*, which can be characterized, e.g., by unique body shape and vivid red body coloration, is a perfect model species for at least some ecological studies including actual habitat preferences and phenological activity. Moreover, we provide one more proof that citizen scientific data can be successfully used not only to analyze present-day ecological parameters of species but also allow to predict its future distribution and response on climate change. In addition, such data can be important addition to discussion about taxonomical status of the studied subspecies.

## Figures and Tables

**Table 1:** Pearson correlation coefficient for 19 bioclimatic variables.

**bio19**	0.496	0.550	0.640	0.415	0.591	0.179	0.465	0.433	0.549	0.599	0.318	0.930	0.898	0.859	0.390	0.910	0.873	0.720	x
**bio18**	0.553	0.826	0.824	0.760	0.848	0.252	0.802	0.819	0.380	0.845	0.353	0.916	0.913	0.859	0.691	0.912	0.870	x	
**bio17**	0.527	0.636	0.684	0.537	0.686	0.165	0.582	0.582	0.457	0.701	0.291	0.951	0.866	0.997	0.394	0.875	x		
**bio16**	0.561	0.757	0.805	0.656	0.785	0.245	0.704	0.688	0.490	0.782	0.368	0.979	0.997	0.857	0.657	x			
**bio15**	0.444	0.863	0.820	0.894	0.827	0.244	0.908	0.766	0.342	0.773	0.347	0.556	0.672	0.376	x				
**bio14**	0.509	0.619	0.664	0.527	0.668	0.152	0.569	0.564	0.441	0.683	0.274	0.938	0.849	x					
**bio13**	0.567	0.763	0.810	0.661	0.790	0.258	0.709	0.698	0.492	0.787	0.381	0.973	x						
**bio12**	0.568	0.724	0.774	0.618	0.764	0.215	0.667	0.660	0.490	0.770	0.343	x							
**bio11**	0.836	0.411	0.539	0.123	0.487	0.838	0.199	0.535	0.779	0.555	x								
**bio10**	0.806	0.951	0.944	0.760	0.991	0.364	0.833	0.936	0.612	x									
**bio9**	0.816	0.508	0.625	0.188	0.569	0.551	0.275	0.441	x										
**bio8**	0.725	0.891	0.860	0.744	0.923	0.381	0.803	x											
**bio7**	0.398	0.919	0.829	0.990	0.891	0.102	x												
**bio6**	0.592	0.234	0.355	0.056	0.308	x													
**bio5**	0.741	0.981	0.958	0.826	x														
**bio4**	0.297	0.856	0.753	x															
**bio3**	0.751	0.961	x																
**bio2**	0.656	x																	
**bio1**	x																		
	**bio1**	**bio2**	**bio3**	**bio4**	**bio5**	**bio6**	**bio7**	**bio8**	**bio9**	**bio10**	**bio11**	**bio12**	**bio13**	**bio14**	**bio15**	**bio16**	**bio17**	**bio18**	**bio19**

**Table 2:** Relative contributions of the environmental variables to the Maxent model.

*Cucujus clavipes clavipes*		*Cucujus clavipes puniceus*	
Variable	Percent Contribution	Variable	Percent Contribution
bio1	44.3	bio1	35.9
bio12	23.9	bio9	20.2
srad07	7.8	land cover	10.6
srad12	7.3	bio10	10.4
bio6	4.3	soil class	5.6

**Table 3:** Future changes in coverage of suitable niches of studied taxa [km^2^].

***Cucujus clavipes clavipes***					
scenario	−1 (range expansion)	0 (absent in both)	1 (present in both)	2 (range contraction)	Change
ssp126	92,386.29	19,997,564	204,237.6	788,769.7	−70.13%
ssp245	518,292.9	19,571,658	192,326.2	800,681.2	−28.44%
ssp370	569,134.4	19,520,816	159,206.6	833,800.8	−26.65%
ssp585	580,755.3	19,509,195	133,059.5	859,947.8	−28.12%
***Cucujus clavipes puniceus***					
scenario	−1 (range expansion)	0 (absent in both)	1 (present in both)	2 (range contraction)	Change
ssp126	109,236.6	20,422,890	210,338.6	340,492.9	−41.98%
ssp245	161,530.7	20,370,596	175,766.4	375,065.1	−38.77%
ssp370	213,824.9	20,318,301	191,454.6	359,376.9	−26.42%
ssp585	218,763.8	20,313,363	72,630.73	478,200.7	−47.10%

## Data Availability

Not applicable.

## Supplementary Materials

The following are available online at https://www.mdpi.com/article/10.3390/insects12040369/s1, Data S1: Records of *C. c. clavipes* and *C. c. puniceus* accepted for this study from the iNaturalist.org database shown according to the scheme: latitude/longitude/(day.month.year). Records marked with asterisk (*) were accepted for the ENM analysis.
