# Supplementary material for: From Phenology and Habitat Preferences to Climate Change: Importance of Citizen Science in Studying Insect Ecology in the Continental Scale with American Red Flat Bark Beetle, Cucujus clavipes, as a Model Species
Source: Insects. 2021 Apr 20;12(4):369. doi: 10.3390/insects12040369 (PMC8074780; doi:10.3390/insects12040369)
Supplement: Supplementary file 1 [file insects-12-00369-s001.zip › insects-1173004-supplementary.pdf]

Data S1. Records of *C. c. clavipes* and *C. c. puniceus* accepted for this study from the iNaturalist.org database shown according to the scheme: latitude/longitude/(day.month.year). Records marked with asterisk (*) were accepted for the ENM analysis.

Cucujus clavipes clavipes:

CANADA: ***Manitoba***: *49.837759/−97.079408 (14.04.2015); 50.241601/−97.30696 (22.04.2016); 49.422937/−96.275963 (29.04.2018); 49.873046/−97.105548 (14.04.2019); *49.92032/−99.612687 (30.04.2020); 50.552116/−99.771591 (07.04.2020); 50.08747/−96.859134 (21.05.2020); ***New Brunswick***: *46.390469/−64.620765 (10.05.2018); 46.052077/−64.929756 (14.10.2019); *45.995173/−67.238639 (15.05.2020); *45.72185/−65.498251 (03.05.2020); 45.960541/−66.64727 (27.08.2020); ***Nova Scotia***: *45.010064/−63.571225 (25.04.2018); *45.95113/−61.169097 (19.05.2019); ***Ontario***: *45.361712/−79.282612 (27.03.2007); 44.590922/−79.539184 (06.04.2012); *42.078799/−82.4928 (13.04.2014); *42.982784/−81.283909 (12.04.2015); *43.743961/−79.801733 (16.04.2016); 43.538983/−80.215618 (09.04.2017); 42.29887/−81.842491 (24.10.2017); *45.513108/−80.319687 (27.10.2017); 43.39558/−80.4739 (30.04.2018); *45.385296/−75.22712 (21.04.2018); 45.209885/−75.226715 (23.04.2018); 45.183483/−76.124261 (27.09.2018); 41.963064/−82.518997 (27.04.2018); *45.247927/−77.994747 (22.12.2018); 44.902939/−76.095801 (27.04.2018); 43.86914/−79.310987 (26.04.2018); 44.722866/−78.26313 (18.09.2019); 44.054967/−80.262297 (22.10.2019); 43.528196/−80.227672 (02.04.2019); 42.948984/−81.429989 (18.06.2019); 43.535987/−80.247819 (15.04.2019); 43.882929/−79.025355 (20.03.2019); 44.507813/−76.55098 (18.03.2019); 43.954519/−79.081029 (21.09.2019); 44.026753/−79.535996 (21.09.2019); 42.985944/−81.253332 (08.10.2019); 42.270889/−81.854967 (19.07.2019); 45.383568/−79.277397 (12.10.2019); 43.576561/−79.999206 (16.05.2020); 45.220432/−75.721315 (12.03.2020); 43.041381/−81.277843 (04.04.2020); 45.274263/−79.284058 (17.05.2020); 43.694404/−80.249126 (06.03.2020); 44.042914/−80.292243 (09.03.2020); 43.696847/−80.245129 (20.03.2020); *45.098956/−81.404255 (12.04.2020); 43.694519/−80.249175 (06.03.2020); 43.69686/−80.245402 (27.03.2020); 42.670939/−80.404099 (08.04.2020); 43.769979/−79.147862 (11.04.2020); 43.867358/−80.763091 (03.05.2020); 42.96365/−81.153571 (27.05.2020); 45.199712/−79.002442 (03.02.2020); 45.356236/−75.677017 (07.06.2020); 42.897259/−78.949552 (12.05.2020); 44.559937/−79.439562 (24.05.2020); 43.477867/−80.770845 (16.05.2020); *46.663597/−84.259767 (06.05.2020); 45.349924/−75.823613 (17.05.2020); *43.967787/−78.676889 (17.03.2020); 44.71798/−78.997725 (02.04.2020); 43.827297/−79.410697 (26.05.2020); 43.033677/−81.231837 (03.05.2020); 42.092625/−82.442372 (24.05.2020); 43.812048/−79.15372 (16.06.2020); 44.586376/−79.51429 (13.05.2020); 42.263861/−83.074863 (23.03.2020); 42.26292/−83.079179 (20.03.2020); 43.185465/−80.657084 (03.05.2020); ***Quebec***: *48.414036/−71.037335 (10.06.1986); *45.418156/−72.681962 (06.06.2008); *46.8361/−71.340974 (03.05.2013); 46.793919/−71.30717 (10.09.2017); 46.927472/−71.194595 (06.05.2017); 46.966537/−71.303596 (13.05.2017); 46.792101/−71.327213 (27.08.2017); *45.967045/−74.159461 (09.05.2018); *48.31783/−69.422046 (10.10.2019); 45.363342/−72.202098 (29.09.2019); 46.775329/−71.287033 (13.10.2019); 45.463641/−74.270821 (29.10.2019); 48.400019/−71.039766 (06.09.2019); 46.809792/−71.295425 (12.09.2019); 46.838392/−71.247569 (27.06.2020); 45.58007/−74.383804 (29.05.2020); 46.810574/−71.315289 (11.05.2020); 45.463641/−74.270821 (03.04.2020); 46.806267/−71.306075 (15.05.2020);

USA: ***Alabama***: *34.726026/−86.641047 (07.11.2017); 34.725994/−86.641241 (07.11.2017); *33.180937/−87.024261 (30.11.2019); ***Arkansas***: *35.687298/−90.39914 (23.02.2020); ***Connecticut***: 41.677455/−73.291488 (04.11.2019); *41.373005/−72.572137 (27.03.2020); 41.725463/−73.209599 (28.04.2016); ***Delaware***: 39.787892/−75.619263 (08.04.2019); ***District of Columbia***: *38.970879/−77.043578 (06.04.2019); 38.974474/−77.046453 (30.03.2019); 38.975997/−77.043454 (06.07.2019); 38.970612/−77.044115 (16.04.2017); 38.97093/−77.043475 (27.06.2020); 38.937496/−77.067305 (06.06.2020); 38.973322/−77.047269 (19.07.2014); 38.973322/−77.047269 (12.03.2016); 38.973806/−77.047934 (10.09.2016); 38.977368/−77.044233 (07.09.2017); 38.973064/−77.046432 (07.09.2014); 38.974665/−77.039372 (09.10.2016); 38.97/−77.04 (28.07.2018); 38.986357/−77.049861 (22.06.2019); 38.97945/−77.043593 (08.06.2019); 38.970834/−77.043046 (28.04.2019); 38.977515/−77.041104 (28.09.2018); ***Illinois***: *41.994045/−87.765213 (01.03.2020); 41.213688/−88.025917 (26.04.2019); 41.21369/−88.026056 (24.04.2019); 41.212788/−88.013835 (26.04.2019); *42.298923/−90.10312 (15.06.2018); 42.1114/−88.292512 (06.04.2019); 41.638233/−87.975137 (08.04.2019); *37.484444/−89.350441 (13.04.2018); 42.428842/−87.931395 (20.04.2014); 41.680038/−87.863 (21.02.2017); ***Indiana***: *39.230371/−87.045844 (14.02.2020, 21.02.2020); *39.949812/−85.872162 (12.04.2020); 39.108567/−86.582414 (06.04.2020); ***Kansas***: *38.978655/−95.239913 (14.09.2019); 38.409415/−94.660177 (20.03.2017); ***Kentucky***: *36.699034/−84.472586 (29.03.2017); *38.960138/−84.54815 (28.04.2020); *37.946456/−83.796999 (10.03.2020); ***Maine***: *44.098075/−70.286614 (03.05.2020); *43.600989/−70.234141 (15.05.2020); *44.492947/−68.996871 (30.05.2018); 44.500895/−68.955724 (16.11.2019); *43.211814/−70.67791 (16.11.2017); ***Maryland***: 38.617278/−76.803434 (17.05.2020); 39.001403/−76.82225 (25.05.2020); *38.80798/−76.140097 (29.04.2020); *39.715831/−79.341157 (28.03.2020); 39.001403/−76.822272 (04.10.2019); 39.366277/−76.646792

(25.04.2019); 39.132786/−76.909286 (04.04.2019); 39.472828/−76.40162 (19.02.2017); 39.647376/−77.438017 (25.03.2017); 38.874108/−76.550347 (09.03.2017); 39.60553/−76.138853 (15.05.2016); ***Massachusetts***: *42.288197/−71.749725 (10.03.2020); *42.058688/−72.686059 (14.04.2019); 42.099162/−72.711271 (05.10.2019); *41.985802/−70.957245 (04.05.2018); 42.407852/−71.471493 (26.03.2006); ***Michigan***: 42.359546/−82.93111 (08.04.2020); *43.029548/−83.604576 (08.04.2020); 42.709817/−84.335687 (16.05.2020); *44.80399/−83.582214 (04.05.2020); *42.882878/−85.705537 (28.04.2020); *43.785199/−86.433782 (02.05.2020); 42.80747/−84.368722 (20.04.2020); 42.798027/−84.900439 (07.04.2020); 42.94931/−85.549923 (15.11.2019); *45.192132/−85.118897 (09.10.2019); 42.810445/−85.733526 (02.05.2018); 42.63794/−85.296096 (08.04.2019); 44.883467/−85.001893 (01.04.2019); 42.924059/−83.670905 (29.04.2009); 42.406641/−85.38224 (23.05.2018); 43.094655/−84.160974 (30.04.2018); *46.564782/−86.15302 (09.05.2017); *41.95332/−86.045083 (04.05.2009); 42.27203/−83.767167 (13.08.2008); 42.801697/−84.443771 (22.04.2018); 44.61067/−85.519016 (12.06.2017); ***Minnesota***: *44.467647/−93.181604 (18.11.2017); *46.799346/−96.121101 (11.04.2015); 43.955594/−92.439576 (30.05.2020); *46.815507/−92.10008 (10.06.2020); 44.8217/−93.120072 (24.05.2020); 46.770878/−92.198572 (23.05.2020); 44.923994/−92.95938 (20.05.2020); 47.124059/−92.677125 (30.04.2020); 44.970176/−93.234941 (01.05.2020); 44.91544/−93.128012 (27.04.2020); 44.972033/−93.387624 (07.04.2020); 45.198193/−93.22973 (27.03.2020); 43.974687/−93.917817 (20.03.2006); 45.092758/−92.87999 (19.10.2019); 44.637922/−93.409627 (29.10.2019); *46.212361/−93.07313 (07.09.2019); 45.301812/−93.607752 (16.04.2019); 44.397792/−92.101148 (22.10.2018); *47.382497/−91.193567 (18.10.2018); 44.936375/−93.194183 (14.09.2018); 46.923924/−96.746818 (13.05.2018); 44.897903/−93.363361 (16.04.2017); 44.467455/−93.17993 (12.04.2016); 44.898315/−93.362955 (27.11.2015); 44.4677/−93.18037 (07.03.2016); 44.46303/−93.171089 (30.03.2013); ***Missouri***: 39.283803/−94.491897 (11.07.2020); *39.373537/−90.905908 (14.10.2017); ***Nebraska***: *42.205987/−97.237774 (22.06.2020); **New *Hampshire***: *43.22881/−71.421562 (19.04.2020); *43.643112/−72.173662 (22.05.2020); 43.286182/−71.460025 (03.05.2020); 44.086811/−71.919991 (20.10.2019); ***New Jersey***: 39.892764/−75.138551 (14.05.2020); 39.892764/−75.138551 (14.05.2020); 40.744869/−74.552316 (23.04.2015); *40.364539/−74.928361 (17.04.2016); 41.186414/−74.798443 (11.04.2015); 40.744796/−74.550501 (23.04.2015); ***New York***: *41.006673/−73.865337 (05.04.2020); *43.042275/−73.782197 (04.04.2020); 43.054061/−78.873383 (29.03.2020); *42.087051/−73.9437 (12.04.2020); *42.88867/−77.519937 (25.05.2020); *42.434439/−76.486392 (11.04.2020); 42.700725/−78.803905 (26.04.2020); 42.936733/−76.54132 (28.04.2020); 42.444141/−76.755413 (28.04.2020); 41.843918/−75.055129 (02.05.2020); 41.932887/−74.154432 (03.05.2020); 42.447848/−76.808455 (03.05.2020); 43.254866/−75.996267 (12.05.2020); 41.690188/−74.372528 (24.05.2020); 43.267184/−76.43317 (12.05.2020); *40.962598/−73.109007 (14.03.2020); *44.568706/−73.776379 (20.03.2020); 43.028295/−76.133355 (09.03.2020); 42.452415/−76.516609 (20.03.2020); 42.367429/−79.215067 (28.06.2014); 44.852011/−74.278325 (01.06.2019); 42.443508/−76.758202 (06.05.2019); 43.194336/−76.541303 (25.05.2019); 41.183788/−73.678856 (30.03.2019); 43.577112/−73.65455 (09.05.2019); 42.844988/−78.850997 (07.04.2019); 42.453514/−76.477386 (13.04.2019); 42.457401/−73.488758 (18.04.2018); 43.032476/−77.062357 (27.04.2018); 42.063244/−76.161 (27.04.2018); *44.206539/−75.250983 (01.05.2018); 41.690017/−73.898296 (03.05.2018); 42.435733/−79.123783 (25.03.2018); 25.03.2018./−78.646787 (01.03.2018); 41.683938/−73.896517 (18.10.2017); 41.690188/−74.372528 (20.05.2020); 42.866195/−75.661863 (20.04.2017); 43.450048/−76.302342 (27.11.2015); ***North Carolina***: *35.204971/−82.565192 (12.05.2020); 35.706774/−82.58289 (11.03.2020); ***Ohio***: 41.310677/−81.875603 (24.05.2020); 41.186375/−81.742705 (17.05.2020); 40.786489/−81.827175 (12.04.2020); 41.449705/−81.273907 (23.05.2020); *39.87648/−83.31669 (04.04.2020); 41.250162/−81.572234 (03.04.2020); 40.515951/−82.785623 (04.04.2020); 41.704669/−80.988903 (07.04.2020); 41.184163/−81.794976 (07.04.2020); *41.641518/−84.364699 (08.04.2020); 41.228744/−81.512017 (03.04.2020); 41.263398/−81.282086 (04.04.2020); 39.169225/−84.103392 (08.03.2020); 41.581864/−81.29516 (08.03.2020); 40.620258/−82.323718 (03.04.2020); 40.992938/−81.679467 (16.04.2019); 40.151971/−83.029063 (16.03.2015); 41.27374/−81.639709 (16.04.2016); *39.33489/−82.130518 (29.10.2019); 40.057683/−83.794822 (28.07.2019); 41.415288/−81.260334 (19.06.2019); 39.591149/−82.28314 (09.02.2019); 41.33613/−81.457397 (07.04.2019); 41.553372/−83.670533 (07.04.2019); 41.575387/−81.264575 (13.04.2019); 41.65355/−81.203383 (01.05.2018); *41.27961/−80.721283 (07.09.2017); 39.640331/−84.455616 (01.05.2018); 41.154194/−81.831566 (12.04.2018); 41.113664/−82.296481 (22.04.2018); 41.333718/−81.657974 (25.03.2017); 39.342247/−84.475325 (12.04.2015); ***Pennsylvania***: 40.650897/−80.176347 (03.05.2020); 39.780123/−76.288228 (09.05.2020); 40.368237/−80.052353 (11.06.2020); *40.423339/−77.685079 (08.04.2020); 40.528912/−80.213967 (25.04.2020); 40.816131/−77.984897 (05.04.2020); 40.013945/−76.353944 (04.03.2020); 40.536295/−79.872112 (14.11.2019); 40.036921/−75.202412 (26.04.2019); 40.815796/−77.985023 (23.05.2019); *40.650235/−79.508615 (08.05.2019); 41.948588/−80.350838 (07.04.2019); 40.732239/−80.247341 (12.04.2018); *41.123322/−76.566353 (04.11.2015); 39.908389/−75.357343 (14.04.2017); 40.253286/−75.476681

(09.05.2016); 39.992643/−77.121209 (17.09.2016); ***South Dakota***: *43.529322/−96.691471 (07.04.2019); ***Tennessee***: *35.745556/−87.209722 (19.03.2020); *35.789203/−85.007747 (03.03.2020); *35.739067/−83.423739 (19.06.2020); *36.590613/−82.292464 (14.12.2018); 35.04563/−85.30968 (14.01.2017); 34.999364/−85.184775 (09.01.2016); ***Vermont***: 44.32915/−73.107492 (01.05.2020); 44.792574/−72.129356 (18.05.2020); 43.782058/−72.357101 (03.05.2020); 44.17826/−73.370764 (04.05.2020); 42.855488/−72.57225 (03.05.2020); 44.658038/−72.678497 (25.04.2020); 44.152275/−72.784242 (19.04.2020); 44.417621/−73.104517 (04.04.2020); 43.831136/−72.63689 (21.03.2020); 44.397317/−72.719125 (20.09.2019); 44.107016/−72.861197 (05.11.2019); 44.391109/−72.966705 (17.05.2019); 43.49038/−72.422375 (13.04.2019); 44.201603/−73.047844 (29.05.2018); 44.580387/−73.198022 (01.06.2017); 43.492743/−72.802447 (07.10.2018); 44.346988/−72.990508 (24.04.2018); 43.59333/−72.452515 (12.05.2016); 44.763536/−72.147131 (11.11.2013); 43.853296/−72.589057 (18.05.2013); ***Virginia***: 38.914532/−77.316994 (11.11.2019); 39.05732/−77.330231 (06.11.2019); 38.907895/−77.114747 (26.04.2020); 38.963904/−77.330996 (13.05.2020); 38.871104/−77.140412 (02.05.2020); 38.871329/−77.141023 (02.05.2020); 38.95726/−77.331575 (11.03.2020); 38.964416/−77.331134 (29.02.2020); 38.960968/−77.313889 (24.02.2020); 38.96447/−77.260842 (20.03.2020); 39.036892/−77.306915 (16.02.2020); 39.009853/−77.311951 (15.02.2020); 38.691218/−77.498949 (24.10.2019); 38.966236/−77.302109 (07.01.2020); 38.957558/−77.312714 (07.02.2020); 38.363955/−77.592987 (03.02.2020); 38.766578/−77.315899 (28.12.2019); 39.040634/−77.310844 (30.09.2019); 38.97724/−77.357078 (30.12.2018); 38.931831/−77.323799 (11.03.2019); 38.932598/−77.321487 (02.12.2018); 38.914772/−77.318802 (05.12.2018); 38.961778/−77.37725 (25.01.2019); 38.371407/−77.332551 (28.02.2016); *39.022483/−78.018816 (08.03.2016); *36.811913/−80.354809 (18.09.2015); *37.608756/−77.373314 (30.11.2014); ***West Virginia***: *38.628336/−79.712122 (06.06.2020); 39.248527/−80.380513 (03.05.2020); 39.4173/−79.118767 (26.03.2020); ***Wisconsin***: *45.487583/−91.619604 (26.04.2020); *43.906778/−88.666971 (08.04.2020); *43.289725/−89.600905 (03.04.2020); 44.522011/−87.993927 (08.04.2017); 44.506429/−87.945322 (09.05.2020).

Cucujus clavipes puniceus:

CANADA: ***Alberta***: *56.737927/−118.279807 (14.05.2009); *56.770201/−111.460096 (22.04.2019); *55.509515/−114.928995 (09.05.2019); *53.550176/−116.768726 (27.03.2020); 56.1425/−117.733611 (28.03.2020); *53.682409/−112.824863 (18.06.2020); ***British Columbia***: *52.431232/−131.373962 (13.06.2009); *49.235814/−123.197508 (13.05.2014); *54.392191/−126.66039 (28.04.2016); *50.11187/−117.898648 (10.05.2017); *56.228209/−120.803791 (27.04.2018); *50.829727/−118.9747 (01.05.2018); 54.392278/−126.66043 (15.05.2018); 49.282562/−122.772483 (09.10.2018); *49.315388/−122.440522 (19.05.2018); 49.526561/−124.699014 (30.08.2019); *49.589283/−125.533539 (09.06.2019); *54.066723/−122.985928 (10.05.2019); *48.926228/−123.455338 (04.05.2019); 49.314076/−122.748429 (09.05.2019); 49.248506/−123.219174 (12.05.2019); 50.660482/−119.164947 (26.05.2019); 49.594313/−125.530121 (09.06.2019); *49.779522/−123.1167 (02.06.2019); *52.505713/−121.226131 (09.05.2019); *49.511311/−115.05632 (14.05.2019); *52.337658/−125.769883 (18.06.2019); *50.519985/−122.845314 (10.04.2020); 49.826163/−125.051864 (24.04.2020); *48.464984/−123.319414 (06.04.2020); *48.766623/−123.887618 (05.05.2020); 49.335833/−122.768333 19.04.2020); 49.466956/−123.237139 (08.05.2020); 49.466954/−123.237015 (08.05.2020); 49.314723/−122.915475 (08.05.2020); 49.320563/−122.735607 (30.05.2020); 50.276047/−119.216238 (10.05.2020); *54.519995/−128.539837 (04.05.2020); 49.440676/−123.437167 (10.05.2020); 50.516012/−122.720571 (08.05.2020); 53.51348/−123.275903 (20.06.2019); 49.552533/−117.52055 (04.07.2020); *49.101971/−121.92378 (18.04.2020); *49.917481/−116.916377 (09.05.2020); 49.518208/−123.213487 (19.07.2020); 50.558922/−122.277905 (21.07.2020); *49.189308/−125.287835 (25.07.2020); 49.535095/−121.877822 (03.06.2020);

USA: ***Alaska***: *57.053583/−135.327256 (09.01.2013); *60.458659/−151.082085 (29.12.2014); 57.054071/−135.326873 (28.05.2015); *57.092324/−135.450436 (05.07.2015); *58.29773/−134.384247 (10.06.2016); 60.714008/−151.366058 (22.05.2017); *61.019093/−149.480074 (27.04.2018); 61.229973/−149.26973 (07.04.2018); 57.049998/−135.321586 (06.05.2018); 58.407403/−134.583953 (12.06.2018); 61.154438/−150.063278 (26.05.2018); 61.310188/−149.503045 (03.04.2019); 61.164055/−149.764984 (26.04.2019); 61.589683/−149.099883 (17.04.2019); 60.529235/−150.819481 (15.04.2019); *56.36096/−132.3557 (27.04.2019); 60.701212/−151.321547 (25.05.2020); *60.544029/−145.75798 (05.06.2020, 15.06.2020); *64.904448/−147.782007 (29.04.2020, 02.05.2020); *58.131383/−135.465605 (09.05.2020); ***California***: *34.147385/−116.982348 (04.03.2020); 37.092214/−119.231353 (26.06.2020); *41.652407/−123.798178 (31.05.2020); *39.210983/−120.812912 (22.05.2020); *36.58477/−118.705903 (24.04.2020); 37.081695/−119.315767 (24.04.2020); *40.316553/−121.183502 (13.04.2020); 36.584642/−118.705772 (24.04.2020); 38.589516/−121.458215 (31.03.2020); 39.432115/−120.24123 (25.06.2016); 39.432137/−120.241228 (25.06.2016); *38.734335/−122.758934 (19.03.2017); *37.991325/−120.241352 (15.02.2020); *37.609817/−119.024024 (09.07.2019); 36.5815/−118.751563 (20.06.2019); 38.920775/−120.657608 (29.05.2019); 36.740917/−118.974237 (25.07.2019); 39.863872/−120.16389

(12.06.2019); 38.025561/−119.971337 (03.06.2019); 37.113079/−119.293276 (06.06.2019); 37.074117/−119.344475 (11.06.2019); 39.405142/−123.790839 (07.04.2019); 37.753312/−119.837937 (20.04.2019); 36.727962/−118.979753 (21.06.2018); 36.562653/−118.785208 (05.05.2018); ***Colorado***: *39.747192/−104.947692 (20.03.2018); *38.771382/−104.651917 (11.03.2020); 39.978358/−105.110703 (07.03.2020); 40.058697/−105.158768 (07.03.2020); 38.678499/−104.699997 (17.02.2017); 40.547255/−104.69507 (24.04.2019); *40.088861/−102.227526 (08.04.2017); ***Idaho***: *43.93713/−115.62212 (26.05.2017); *48.735835/−116.859828 (18.05.2019); *42.751196/−112.393959 (29.04.2020); 42.749167/−112.393889 (29.04.2020); *46.943462/−116.412513 (10.05.2020); ***Montana***: *46.876666/−114.013835 (27.06.2020); 46.876175/−114.013044 (22.05.2020); 46.875825/−113.974663 (11.05.2019); *45.198259/−109.243551 (18.04.2019); *45.404113/−111.222625 (28.05.2018); *48.20621/−114.26109 (22.04.2017); 48.680557/−113.818359 (10.06.2016); ***New Mexico***: *34.431597/−104.211099 (25.01.2020); 34.43162/−104.21122 (28.06.2019); 34.431035/−104.219247 (14.01.2016); ***Oregon***: *44.404827/−121.730599 (25.05.2020); *42.237489/−122.766243 (05.01.2020); *44.048259/−123.049263 (25.05.2020); *45.281647/−121.684988 (04.07.2020); 45.383571/−121.58475 (11.07.2020); *45.416187/−118.125558 (29.05.2020); *43.535707/−123.94188 (29.05.2020); 45.170386/−121.576572 (10.05.2020); *45.200958/−123.723381 (18.04.2019); *44.400741/−119.329268 (05.05.2019); 44.536685/−123.249831 (03.06.2018); 45.493458/−121.814172 (21.05.2018); 44.046856/−124.098068 (05.05.2018); *43.722328/−121.192108 (03.06.2017); 44.052861/−121.26928 (17.11.2017); 42.899965/−122.095533 (22.07.2016); ***Utah***: *41.770367/−111.764917 (29.04.2020); *40.5/−112.06 (29.04.2020); ***Washington***: *48.702413/−122.483723 (14.04.2020); *47.478538/−121.76075 (09.04.2020); *47.572002/−117.404108 (02.04.2020); *46.939676/−121.685755 (06.07.2010); *47.196195/−122.476142 (28.03.2020); 47.782625/−117.498756 (06.03.2020); 47.428328/−122.501949 (30.05.2012); 47.195357/−122.86348 (04.04.2019); *47.592167/−123.82238 (16.06.2016); 46.752828/−121.814362 (30.06.2019); *47.709/−121.13813 (01.07.2019); 47.072632/−121.578613 (09.06.2019); 47.961867/−123.180243 (18.05.2019); *48.774088/−119.29715 (23.04.2019); 47.972206/−121.998391 (30.03.2019); *46.276498/−121.59918 (18.07.2018); *48.699398/−123.014617 (13.04.2008); *47.210348/−120.941264 (20.06.2020); *46.146636/−122.315628 (10.05.2020); 47.660735/−121.203572 (19.06.2020); 47.413494/−121.426142 (28.05.2020); *47.906784/−122.297592 (11.05.2020); 48.751911/−122.478686 (15.05.2020); 47.823084/−122.369647 (05.05.2020); 47.696944/−122.050278 (05.05.2020); 48.664658/−122.332792 (29.04.2020); 48.528576/−122.201818 (16.04.2017); 46.902954/−121.63891 (04.07.2017); 46.079151/46.079151 (03.06.2017); 48.528576/−122.201818 (16.04.2017); 47.013514/−122.497857 (26.04.2020); 47.523359/−122.045724 (17.04.2020); *47.751653/−123.078757 (15.07.2020); 47.696356/−122.211995 (12.04.2020); 47.696405/−122.211804 (12.04.2020); 47.696295/−122.211543 (12.04.2020); 47.696214/−122.211961 (11.04.2020); 47.696296/−122.211546 (12.04.2020); 46.88105/−121.590415 (31.05.2020); 48.882512/−121.534705 (21.07.2020); ***Wyoming***: *43.478907/−110.884962 (07.05.2020); 43.479096/−110.884724 (07.05.2020); *44.956893/−106.986544 (06.04.2019); *41.315563/−105.589312 (04.03.2018).
